# Laser controlled atom source for optical clocks

**DOI:** 10.1038/srep37321

**Published:** 2016-11-18

**Authors:** Ole Kock, Wei He, Dariusz Świerad, Lyndsie Smith, Joshua Hughes, Kai Bongs, Yeshpal Singh

**Affiliations:** 1 School of Physics and Astronomy, University of Birmingham, Edgbaston Park Road, Birmingham B15 2TT, UK

## Abstract

Precision timekeeping has been a driving force in innovation, from defining agricultural seasons to atomic clocks enabling satellite navigation, broadband communication and high-speed trading. We are on the verge of a revolution in atomic timekeeping, where optical clocks promise an over thousand-fold improvement in stability and accuracy. However, complex setups and sensitivity to thermal radiation pose limitations to progress. Here we report on an atom source for a strontium optical lattice clock which circumvents these limitations. We demonstrate fast (sub 100 ms), cold and controlled emission of strontium atomic vapours from bulk strontium oxide irradiated by a simple low power diode laser. Our results demonstrate that millions of strontium atoms from the vapour can be captured in a magneto-optical trap (MOT). Our method enables over an order of magnitude reduction in scale of the apparatus. Future applications range from satellite clocks testing general relativity to portable clocks for inertial navigation systems and relativistic geodesy.

In recent years, there has been tremendous progress in the field of precision metrology and quantum measurements with optical lattice clocks demonstrating a frequency uncertainty on the order of 2 parts in 10^18^ 1,2,3,4. In an optical lattice clock, many thousands ultracold neutral atoms confined in periodic light potentials can be used simultaneously for precision metrology and quantum measurements, and the spectroscopy becomes independent of the motion of the centre-of-mass of the atoms^5^. The wavelength and polarisation of the lattice laser is chosen such that the two probed electronic states of an atom experience the same light shifts and perturbations on the clock transition are minimised. One central remaining systematic effect in these systems is posed by systematic shifts caused by black body radiation (BBR)^6^,^7^,^8^. This thermal radiation depends on the temperature of the enclosure of the probed atoms, where particular challenges result from an inhomogeneous thermal distribution around the atom source. This is of particular importance for Sr, which typically has to be heated to several hundred degrees to generate sufficient vapour pressure.

The atom source creates another limitation when it comes to novel applications of state-of-the-art clocks. The typically used Zeeman slower systems add size, complexity and power consumption, rendering the creation of a master space optical clock^9^,^10^ or portable clocks for mapping the Earth’s gravitational field via relativistic geodesy^11^,^12^,^13^ extremely challenging.

Here we report on a novel atom source, which operates without any significant heat generation, uses minimal space and allows a high degree of control in a simple, low-power setup^14^. Starting from strontium-oxide, our source differs from light-induced atomic desorption (LIAD)^15^,^16^,^17^,^18^,^19^,^20^,^21^, which relies on desorption of the metal itself. LIAD has been successfully used with alkali metals, but to our knowledge has not been demonstrated for alkaline earth metals. We demonstrate the practicality of our laser-controlled atom source by the operation of a Sr magneto-optical trap (MOT) and we characterise lifetimes and velocity distributions. We find that the parameters are suitable for the operation of optical lattice clocks, opening the pathway to compact systems for novel applications outside of the laboratory.

## Results

**Figure 1:**
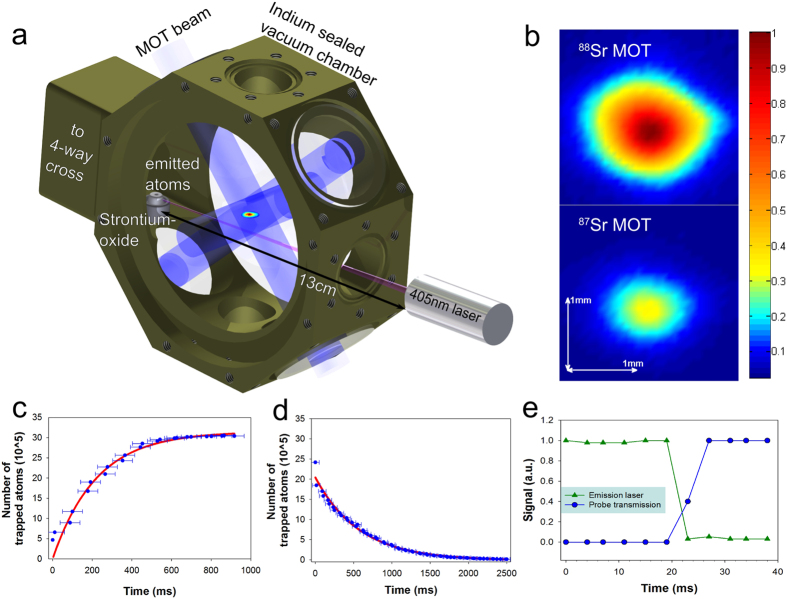
Experimental setup. (**a**) A schematic showing all three pairs of MOT beams, the emission laser at 405 nm and the vacuum chamber. The figure shows a low power diode laser at 405 nm focused onto a strontium oxide sample in solid form. As a result, Sr atoms are emitted and captured in a 3D-MOT. The MOT beams are shown in blue. The MOT fluorescence and a separate probe beam at 461 nm (not shown here) are used as the two detection methods for the Sr vapours. (**b**) Typical photographs of a ^88^Sr (top) and ^87^Sr (bottom) MOT, consisting of ~4 × 10^6^ atoms and ~1 × 10^6^ atoms, respectively. (**c**) Loading of the ^88^Sr MOT. (**d**) Decay of the ^88^Sr MOT. (**e**) Measured delay between switching off the emission laser and Sr vapour emission. After switching off the emission laser, an increase in the probe beam transmission is detected. In all the panels, filled circles represent data while solid lines (red colour) represent exponential fits to the data. A loading and decay time of ~200 ms and ~500 ms, respectively, are measured.

**Figure 2:**
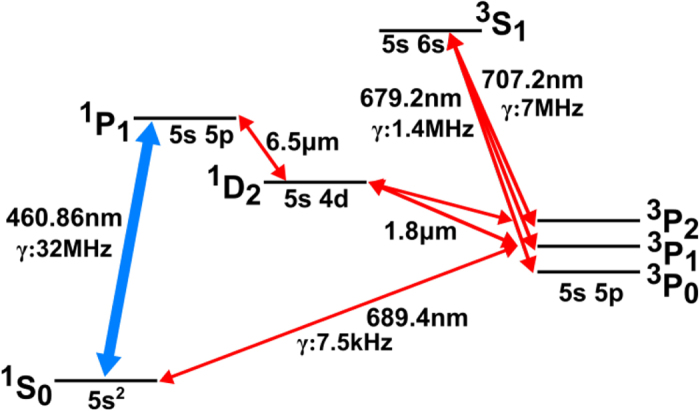
Energy level diagram of Sr. We show only the energy levels relevant to MOT operation. γ refers to the transition linewidth, and ^1^S_0_-^1^P_1_ is the main cooling transition. A small fraction of atoms can be lost to ^3^P states via the ^1^D_2_ state (see text).

Our atom source relies on the release of Sr atoms from a bulk sample of strontium oxide when irradiated with a laser. In the experimental setup shown schematically in Fig. 1a, we employ a simple off-the-shelf 405 nm diode laser module for this purpose. However, we also observe atom emission for other wavelengths, including 532 nm, 922 nm and 1560 nm, at laser powers as low as 5 mW. When irradiating the SrO, we can readily see fluorescence of Sr atoms in a nearby probe beam resonant with the 461 nm transition in Sr (Fig. 2). We anticipate that in addition to Sr, also oxygen will be released to the vacuum chamber, and we observe a spike in pressure on the ion pump when the sample is illuminated. We typically observe an increase of the vacuum pressure from approx. 1 × 10^–9^ mbar to ~1 × 10^–8^ mbar, when the emission laser is switched on. The pressure is inferred from the ion pump current and is in good agreement with the life-time measurements of our blue MOT (see below). It is important to consider appropriate pumping geometries to allow a quick reduction of background gas pressure, after capturing the desired Sr atoms. The remaining question is whether the velocity profile of the emitted atoms contains suitably low velocities to allow trapping with laser cooling methods.

To demonstrate the applicability of our source to a cold atom experiment, we trap the emitted Sr atoms in a MOT. While the data in the remainder of this paper is based on measurements using the ^88^Sr isotope, we are also able to load a MOT using other Sr isotopes, in particular ^87^Sr, which is most commonly used in Sr optical lattice clocks (Fig. 1b).

Figure 1c shows a typical loading curve leading to ~3 × 10^6^ trapped ^88^Sr atoms (Fig. 1b) without much optimisation. At the resulting density of 4 × 10^9^ atoms per cm^3^, collisions inside the MOT are insignificant^22^, allowing a fit of the loading and decay curves in Fig. 1c,d with exponential functions. The resulting 1/*e* loading and decay times are ~200 ms and ~500 ms, respectively. The difference is due to the rapid drop of background gas pressure when switching off the emission laser, leading to longer decay times. This is supported by the observation that Sr fluorescence measurements show a fast drop to zero when the emission laser is switched off (Fig. 1e). The trapped atom number and loading and decay rates can be optimised by adapting the geometry of the setup and changing laser power, focus and pulse duration. We have been able to trap ~5 × 10^5^ atoms for emission laser pulses of ~200 ms, and one can anticipate achieving a higher number of trapped atoms for shorter duration of the emission laser by reducing the distance between the sample and the MOT centre.

### Velocity distribution

**Figure 3:**
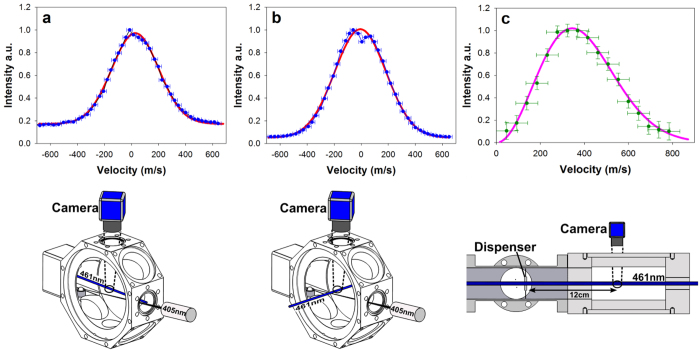
Velocity distribution of the emitted atoms. A probe beam at 461 nm was used to detect the fluorescence of the emitted atoms (y-axis). The velocity on the x-axis corresponds to the Doppler detuning of the probe beam. (**a**) Probe beam is nearly antiparallel to the emission laser. (**b**) Retroreflected probe beam orthogonal to the emission beam with a Lamb-Dip visible in the centre. The small asymmetry in the distribution is caused by Doppler effect due to the emission of atoms from the source which is slightly off axis of the chamber geometry. (**a**,**b**) The filled circles represent the data while the solid lines represent Gaussian fits to the data. The data in both curves has been independently rescaled. (**c**) Thermal emission of Sr atoms from a traditional dispenser.

In order to further understand the process, we have measured the velocity distribution of the emitted Sr atoms using Doppler sensitive spectroscopy (Fig. 3). Independently rescaled fluorescence yields have been plotted as a function of velocity of the emitted atoms. Figure 3a shows the velocity distribution of atoms in relation to a probe beam counterpropagating with the emission laser. The nearly symmetric distribution can be well fit with a Gaussian distribution centred on zero velocity and a temperature of 319 K. This indicates that the atoms undergo several collisions and thermalise with the walls of the vacuum chamber. This is surprising, as the low room temperature vapour pressure of strontium would suggest that the atoms should stick to the wall.

**Figure 4:**
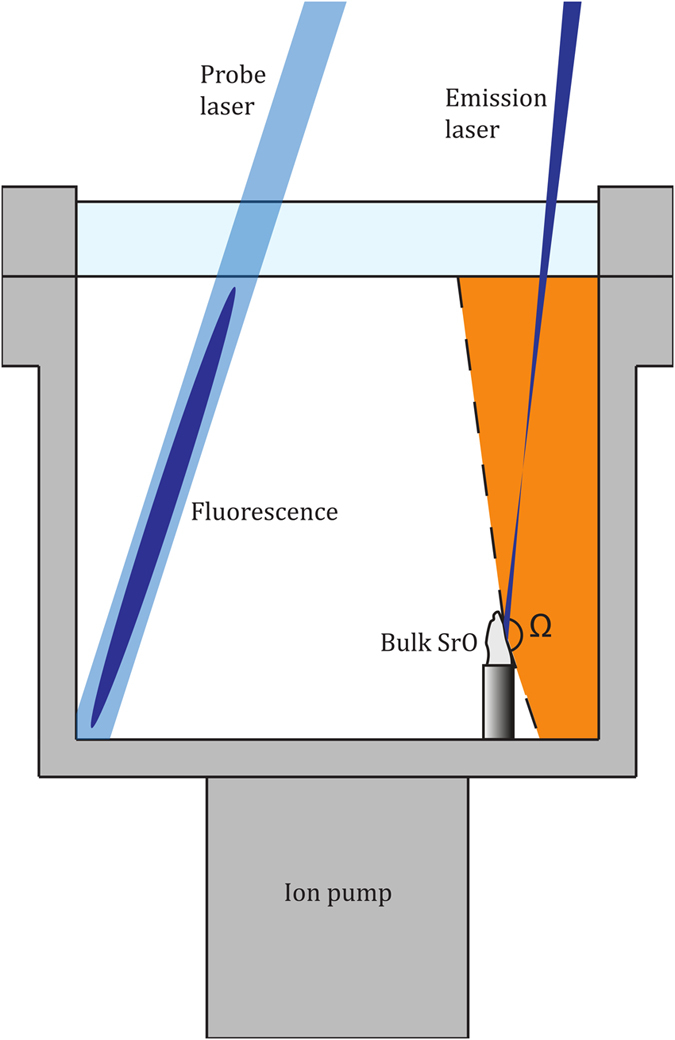
Bongs Observation of fluorescence with no direct line of sight of Sr vapour emission. Experimental setup used to demonstrate that emitted Sr atoms collide with room temperature walls of a vacuum chamber without sticking. At the location where the emission laser hits the SrO sample, the Sr vapour will be emitted in a solid angle, Ω (the orange shaded region in the figure), that does not intersect any point with the beam path of the probe laser. When the emission laser was turned on, however, fluorescence could still be observed in the probe beam, indicating the presence of atomic vapour even after collisions with the chamber walls.

To confirm that atoms are able to reflect off the walls of the vacuum chamber, we performed the experiment illustrated in Fig. 4. In this experiment, fluorescence was still observed despite having no direct pathway for emitted Sr atoms to reach the path of the probe beam. We expect that this effect allows thermalisation with the walls of the vacuum chamber, creating a thermal distribution accessible to laser trapping and cooling. This is consistent with evidence that the measured velocity distribution does not depend on emission laser power or wavelength.

A further consistency check is provided in Fig. 3b, representing a measurement with a retroreflected probe beam perpendicular to the emission laser. This measurement shows a Lamb dip indicating the zero-velocity component and has the same width as the distribution in Fig. 3a, indicating a fully thermalised vapour. Geometry, together with the source in the probe chamber, plays an important role in creating the observed thermalised background vapour. In contrast to the previous setup, a measurement of the emission velocity spectrum in a beam-like geometry in Fig. 3c, using a commercially available dispenser (Alvatec Alvasource, Type S, Fig. 2b in ref. 23), shows a very good fit with a Maxwell-Boltzmann distribution for 626 K.

## Discussion

The presented method allows unprecedented control for generating atomic vapours for laser cooling and trapping applications. It does so by breaking chemical bonds in compounds, which have a negligible vapour pressure at ambient temperature. We find that the method is not restricted to strontium oxide, but also works with strontium hydroxide, strontium carbonate and bulk strontium. For all these samples we detect Sr vapour fluorescence in the vacuum chamber. Qualitatively we observe the indications that Sr fluorescence is the strongest for SrO and that all composites seem to lead to more fluorescence than bulk Sr. However, this might be partly linked to sample preparation.

Though a proper understanding of the process requires further detailed investigations, the insensitivity of our measurements to the emission laser’s wavelength suggests that thermal effects might play a role in the emission process. Finite element simulations suggest that this might be possible if the powdery nature of the sample reduces the thermal conductivity by more than a factor of 100 as compared to bulk strontium oxide.

In any case, our method produces no significant heating and promises to overcome size, power and blackbody shift limitations of current Sr optical lattice clock systems. We anticipate this method will work with other species as well, furthering the ongoing efforts to realise quantum technologies that harness pristine quantum properties to deliver breakthroughs in a wide range of applications including quantum clock networks and deep space navigation, GNSS, VLBI, relativistic geodesy, financial markets and the exploration for minerals and oil^9^,^10^,^11^,^12^,^13^,^24^,^25^,^26^.

## Methods

### Sample preparation and science chamber

Sr vapours are generated from bulk strontium oxide near its surface. In order to generate strontium oxide, we use a solid piece of granular strontium (99% trace metals basis) which is exposed to air for 4 hours such that it can react with oxygen in the air to form strontium oxide. The prepared sample is mounted on a sample holder in the science chamber. The science chamber is a compact, titanium vacuum chamber with indium sealed viewports. The viewports are made of BK7 glass and are AR coated for all relevant wavelengths. We place the strontium oxide sample in solid form into the science chamber using a sample holder. The chamber is evacuated using a 20 l/s ion pump and a turbo-molecular pump. The chamber maintains an ultra high vacuum (~5 × 10^–10^ mbar) environment.

### Emission laser

We have mainly used a commercially available 405 nm diode laser (405 nm UV PHR-805T) with 120 mW as the maximum output power. The distance between the laser and the sample is 14 cm. We use a 4.5 mm aspheric lens to focus the laser light 10 cm away from the sample. At the focal point, the 1/*e*^2^ laser beam diameter is 150 μm in the vertical and 200 μm in the horizontal. This allows for continuous emission of Sr. We have observed that at a tighter focus, one can emit large amounts of Sr. However, it lasts only for short durations, typically only a few seconds. At a shallower focus, on the other hand, we do not observe any emission. We use a separate laser beam at 461 nm in order to observe emission of Sr.

### MOT and detection method

We use a six beam MOT, of which three beams are retro-reflected. Each beam is circularly polarised, 1 cm in diameter, 3 mW/cm^2^ in intensity and approximately 56 MHz red detuned from the resonant transition ^1^S_0_-^1^P_1_ at 461 nm (Fig. 2). Reflection and polarisation changes for the reflected beams are achieved, respectively, by focussing the beams onto mirrors and using achromatic quarter wave plates. To bring atoms in the long lived excited states ^3^P_2_ and ^3^P_0_ back to the ground state ^1^S_0_, we use standard repumping transitions ^3^P_0_-^3^S_1_ at 679 nm and ^3^P_2_-^3^S_1_ at 707 nm. For the MOT, we use a magnetic quadrupole field with a gradient of approximately 40 G/cm at the centre. The MOT centre is approximately 4 cm away from the sample.

To detect the emitted Sr atoms, we use the broad ^1^S_0_-^1^P_1_ transition at 461 nm with 32 MHz linewidth. We use a probe beam of 1 mm diameter and 3–5 mW intensity. When Sr atoms are emitted, they interact with the probe beam, and the resulting fluorescence is detected by a CCD camera (pco.pixelfly usb, model PF-M-QE-PIV). We used a 1 ms exposure time for the fluorescence image of the MOT. In addition to the camera, we have used a photodiode for detecting the absorption of Sr atoms by a probe beam. During normal MOT operation, the probe beam is switched off. During loading, the emission laser is ON, while during the decay time, it is OFF.

### Velocity distribution of emission

We use the fluorescence yield at 461 nm to determine the velocity distribution present in the emission. From the Doppler effect, we can estimate the velocity of atoms from the detuning of the probe laser using the following equation:


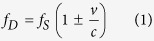


where *f*_*D*_ is the Doppler shifted frequency, *f*_*S*_ is the resonant frequency of the atomic transition, *v* is the velocity component of atoms along the probe beam direction and *c* is the velocity of light. In our Doppler spectroscopy, the velocity of atoms corresponds to the detuning of the probe laser from the resonance at ^1^S_0_-^1^P_1_. We scan the probe frequency by ±2 GHz around the ^1^S_0_-^1^P_1_ transition at 461 nm, while monitoring the wavelength on a wavemeter (ADVANTEST Q8326, 10 MHz resolution). Our apparatus allows us to probe the emitted atoms from two orthogonal directions, one where the probe beam is almost antiparallel to the emission laser (Fig. 1) and the other where the probe beam is orthogonal to the direction of the emission laser.

## Additional Information

**How to cite this article**: Kock, O. *et al*. Laser controlled atom source for optical clocks. *Sci. Rep*. **6**, 37321; doi: 10.1038/srep37321 (2016).

**Publisher’s note**: Springer Nature remains neutral with regard to jurisdictional claims in published maps and institutional affiliations.
